# The effect of biofloc density and *Bacillus* sp. NP5 supplementation on bacterial inhibition, antibiofilm activity, and the immunity of the Pacific white shrimp (*Penaeus vannamei*) against *Vibrio parahaemolyticus*

**DOI:** 10.1016/j.cirep.2025.200228

**Published:** 2025-05-18

**Authors:** Muhamad Gustilatov, Widanarni, Julie Ekasari, Pande Gde Sasmita Julyantoro, Diana Elizabeth Waturangi, Sukenda, Febrina Losiana Silalahi

**Affiliations:** a Department of Aquaculture, Faculty of Fisheries and Marine Sciences, Bogor Agricultural University, Bogor, West Java, 16680, Indonesia; b Department of Aquatic Resources Management, Faculty of Marine Science and Fisheries, University of Udayana, Denpasar, Bali, 80361, Indonesia; c Faculty of Biotechnology, Atma Jaya Catholic University of Indonesia, Jenderal Sudirman No. 51, Jakarta, 12930, Indonesia

**Keywords:** Antibiofilm, Aquatic, Biofloc, Immune response, *Penaeus vannamei*, *Vibrio parahaemolyticus*

## Abstract

This study aimed to evaluate the effect of biofloc density supplemented with *Bacillus* sp. NP5 on bacterial inhibition, antibiofilm activity, growth performance, and the immune response of Pacific white shrimp (*Penaeus vannamei*) against *Vibrio parahaemolyticus*, and to determine the optimal biofloc density for disease resistance in shrimp aquaculture. Biofloc was prepared with molasses as a carbon source (C/N 10) and supplemented with *Bacillus* sp. NP5 (10⁶ CFU/mL). Shrimp (initial weight 0.52 ± 0.16 g) were reared in 33.3 L aquarium at a density of 3000 shrimp/m³ for 21 days, with biofloc densities of 5, 10, and 15 mL/L, and challenged with *V. parahaemolyticus* (10³ CFU/mL). Controls included pathogen-exposed shrimp without biofloc (positive control) and pathogen-free shrimp with no biofloc (negative control). Bacterial inhibition and biofilm formation were assessed, growth performance was measured by final weight and specific growth rate (SGR), and immune responses were evaluated by total hemocyte count, phagocytic activity, respiratory burst, and phenoloxidase activity. While there was no significant difference in growth performance among the biofloc density treatments, the biofloc system overall showed higher growth performance compared to the control groups. However, the 15 mL/L biofloc density significantly reduced *V. parahaemolyticus* density and biofilm formation (*p* < 0.05) and enhanced immune responses compared to the controls. In conclusion, biofloc supplemented with *Bacillus* sp. NP5 significantly improved shrimp health and productivity, with 15 mL/L biofloc density providing optimal pathogen reduction and immune enhancement, despite no significant growth performance differences among the treatments.

## Introduction

The Pacific white shrimp (*Penaeus vannamei*) is one of the most valuable aquaculture commodities globally, contributing approximately 62 % to global crustacean production and accounting for 17 % of the value of aquatic product exports in 2022 [1]. Despite continued growth in production, the industry faces significant challenges, particularly from disease outbreaks such as vibriosis. One of the most prominent diseases is acute hepatopancreatic necrosis disease (AHPND), caused by *Vibrio parahaemolyticus*, which produces PirA and PirB toxins (VPAHPND) [[2], [3]]. AHPND has inflicted considerable economic losses on the shrimp industry, with global losses estimated at USD 44 billion [4]. Moreover, the growing issue of antibiotic resistance in *V. parahaemolyticus* poses a significant challenge to disease management in shrimp aquaculture. Subpopulations of bacteria have formed as a result of antibiotic induction or spontaneous mutations and physical or chemical stresses [5]. This emerging threat highlights the need for alternative approaches, such as the use of biofloc technology, to mitigate bacterial infections effectively without relying on antibiotics.

*V. parahaemolyticus* utilizes several virulence factors to regulate infection, including the type 3 and type 6 secretion systems (T3SS and T6SS), which facilitate attachment to the host and toxin injection [[6], [7], [8]]. Quorum sensing (QS) mechanisms are central to the regulation of these virulence factors, including the expression of PirA and PirB genes linked to AHPND [9,10]. Consequently, strategies to disrupt QS and reduce virulence in pathogenic bacteria have become a critical focus in aquaculture [11].

Biofloc technology is a promising method for controlling pathogenic organisms in shrimp culture [12]. This approach converts nitrogenous waste into heterotrophic microbial biomass by adjusting the carbon-to-nitrogen (C/N) ratio, thereby reducing toxic compounds like ammonia and nitrite while providing supplemental nutrition [13,14]. Biofloc, which consists of bacteria, fungi, algae, and organic matter, enhances water quality, minimizes water usage, and exhibits biocontrol properties, including anti-biofilm and QS-disrupting activities that mitigate pathogenic bacteria like *Vibrio* [[15], [16], [17]].

The use of biofloc in shrimp culture has been shown to prevent *Vibrio* outbreaks, with studies reporting higher survival rates in *P. vannamei* challenged with *V. parahaemolyticus* [18,19]. As biofloc biomass increases during culture, adverse effects such as elevated oxygen consumption and water turbidity may occur, necessitating the regulation of biofloc density. However, biofloc density may also influence its capacity to inhibit pathogenic bacteria. Understanding the optimal biofloc density for bacterial control could provide key insights for microbial management in shrimp farming.

This study aimed to evaluate the effect of biofloc density on bacterial inhibition, antibiofilm activity, and the immune response of the Pacific white shrimp against *Vibrio parahaemo-lyticus*, focusing on biofloc supplemented with *Bacillus* sp. NP5. It examined *V. parahaemolyticus* growth, biofilm formation, and shrimp immune parameters to determine the optimal biofloc density for enhancing disease resistance in shrimp aquaculture.

## Materials and methods

### Experimental design

The study comprised *in vitro* and *in vivo* experiments that followed a completely randomized design. The *in vitro* experiment consisted of seven treatments, while the *in vivo* experiment had five; each experiment was conducted in triplicate. In the *in vitro* experiment, the treatments involved different biofloc densities (5, 10, and 15 mL/L) for both suspension and filtrate. The shrimp receiving these treatments were then exposed to *V. parahaemolyticus* at a density of 10^3^ CFU/mL. The control treatment involved no addition of biofloc suspension nor biofloc filtrate, and neither did it involve a *V. parahaemolyticus* addition. For the *in vivo* experiment, the treatments included three different biofloc densities (5, 10, and 15 mL/L) and a positive control without biofloc, which were challenged with *V. parahaemolyticus* at a density of 10^3^ CFU/mL. A negative control treatment was made without biofloc and challenge.

### Biofloc and bacterial preparation

The biofloc for *in vitro* and *in vivo* analysis was obtained from a biofloc culture stock, which was developed in shrimp culture containers with a working volume of 200 L and a shrimp density of 500 shrimp/m^3^ (shrimp weight 1.5 ± 0.3 g). Molasses, which contains 38 % organic carbon was used as a source of organic carbon, and the culture was maintained for three weeks until the biofloc density reached 20 mL/L. Shrimp in the biofloc culture stock were fed with a commercial feed containing 40–41 % protein; 7 % lipid; 3 % fiber, 13 % ash, and 105 moisture content, four times a day (at 07:00, 12:00, 17:00, and 22:00 Western Indonesian Time) at a feeding rate of 8 %. During maintenance, molasses was added directly into the shrimp rearing container once a day, two hours after the morning feeding, with an estimated C/N ratio of 10. The amount of carbon added was calculated using the carbon requirement calculation scheme by De Schryver et al. [20]. Water quality details in the biofloc culture stock are presented in Table 1. After the biofloc stock volume reached 20 mL/L, the biofloc was diluted according to the volumes used in the treatments. The pathogenic bacteria *V. parahaemolyticus* and probiotic bacteria *Bacillus* sp. NP5 used in this study were obtained from the Laboratory of Health of Aquatic Organisms, Department of Aquaculture, Faculty of Fisheries and Marine Sciences, Bogor Agricultural University. Both groups of bacteria were made resistant in the medium by using 50 mg/mL of rifampicin (0.25 g of rifampicin, 9.5 mL of absolute ethanol, 0.5 mL of distilled water) as a marker (Rf^R^). Thiosulfate-citrate-bile salts-sucrose (TCBS) (Difco, US) medium was used to grow *V. parahaemolyticus*, while seawater complete (SWC) agar was used to grow *Bacillus* sp. NP5 (1 g of yeast extract, 3 mL of glycerol, 5 g of bactopeptone, 250 mL of distilled water, 750 mL of seawater, and 20 g of bactoagar) [21]. The growing colonies were then inoculated in seawater complete broth medium and incubated for 18 h at 28–29 °C using a shaker at a rotating speed of 130 rpm to achieve a bacterial density of 10^9^ CFU/mL. The bacterial density was then diluted until it was appropriate for the challenge test and probiotic supplementation.

**Table 1:** Water quality in the biofloc culture stock.

Parameter	Value
DO (mg/L)	5.5 ± 0.4
pH	7.1 ± 0.2
Salinity (g/L)	32 ± 2
TAN (mg/L)	0.105 ± 0.034
Nitrite (mg/L)	0.379 ± 0.135
Nitrate (mg/L)	0.786 ± 0.228
TSS (mg/L)	287 ± 93

Notes: DO: Disolved oxygen, TAN: total ammoniacal nitrogen, TSS: total suspended solid.

### Shrimp maintenance and *in vivo* challenge test

The animals used in this study were specific pathogen-free Pacific white shrimp (*Penaeus vannamei*) post-larvae (PL 10), obtained from PT. Suri Tani Pemuka hatchery, Anyer, Banten Province, Indonesia. The shrimp were acclimatized and reared until they reached an average body weight of 0.52 ± 0.16 g. Then, the shrimp were placed in a 15-unit glass aquarium with a working volume of 33.3 L and a density of 3000 shrimp per m^3^ for 21 days. During maintenance, commercial feed containing 39–40 % protein was administered four times a day (07.00, 11.00, 15.00, and 20.00 Western Indonesian Time) at a 10 % feeding rate. For the treatments with biofloc, the probiotic bacteria *Bacillus* sp. NP5 at a density of 10^6^ CFU/mL were supplemented into the biofloc system at the beginning of the treatments [22]. The challenge test was performed using *V. parahaemolyticus* Rf^R^ at a concentration of 10³ CFU/mL, which corresponds to the lethal concentration 50 % (LC_50_). One day after the shrimp were transferred to their respective treatment aquaria (both control and biofloc treatments), they were immersed in water containing the bacterial suspension. The bacterial suspension was prepared by culturing *V. parahaemolyticus* in SWC broth and incubating it at 28 °C for 18–24 h to reach the desired concentration, and then the bacterial density was confirmed by plating serial dilutions on TCBS agar. The biofloc density was measured daily using an Imhoff sedimentation cone to maintain the biofloc density according to the treatment. Biofloc was added from the biofloc culture stock to the treatment aquarium that showed a lower biofloc density than the treatment required. Conversely, dilution was carried out using disinfected seawater treated with chlorine at a dosage of 30 µL/L for treatments that exhibited a higher biofloc density than required. Then, the biofloc density was readjusted to match the initial volume. Observations of water quality were carried out on the 1st, 11th, and 21st days of maintenance, focusing on the parameters total ammoniacal nitrogen (TAN), nitrite, nitrate, floc volume, and total suspended solid (TSS). Meanwhile, the parameters dissolved oxygen (DO), pH, and salinity were observed periodically every three days following the Standard Methods for the Examination of Water and Wastewater [23].

### Observation parameters

#### *In vitro* experiment

This experiment measured the effect of biofloc suspension and biofloc filtrate (consisting in the water collected after filtration; Omicron millipore 0.22 µm) on the growth of *V. parahaemolyticus*, as described by Crab et al. [15], using TCBS medium, and anti-biofilm activity, as described by Raissa et al. [24]. A 3 mL aliquot of biofloc suspension or 3 mL of biofloc filtrate for each volume treatment was added to 27 mL of seawater complete medium inoculated with *V. parahaemolyticus* at 10^3^ CFU/mL and then incubated at 28–29 °C for 18 h in a shaker. An anti-biofilm activity assay was carried out by placing a 200 µL sample of bacteria and biofloc suspension or biofloc filtrate in a microtiter plate and incubating it for 24 h at 28–29 °C. After removing the suspension, the plate was rinsed five times with distilled water, air-dried, and then added to 200 µL of 0.4 % (w/v) crystal violet solution. The mixture was incubated for 10 min, washed using distilled water, and air-dried. For biofilm quantification, 100 µL of 95 % ethanol was added to each well to solubilize the bound crystal violet stain, and the plate was incubated for 30 min at room temperature with occasional gentle shaking. The absorbance of the extracted stain was then measured using a microplate reader at an optical density of 650 nm (OD₆₅₀), which represents the biofilm biomass.

#### *Vibrio parahaemolyticus* Rf^R^ density in the shrimp body and rearing medium

The density of *V. parahaemolyticus* Rf^R^ was observed using the Total Plate Count method according to Madigan et al. [25] using the selective thiosulfate-citrate-bile salts-sucrose (TCBS) medium with rifampicin antibiotic (50 μg/mL) on the seventh day after the challenge test. Water samples (1 mL) and mashed shrimp bodies (0.5 g) were serially diluted and spread as much as 50 μl on the medium. Then the medium containing the bacterial culture was incubated for 24 h at 28–29 °C [25]. After incubation, bacterial colonies exhibiting typical Vibrio morphology (yellow or green colonies on TCBS agar) were counted. The bacterial count was determined as colony-forming units per milliliter (CFU/mL) for water samples and colony-forming units per gram (CFU/g) for shrimp tissue samples. The results were recorded and analyzed to assess bacterial load variations between treatments.

### Shrimp growth performance

In this study, the assessed growth performance indicators included specific growth rate (SGR) [26]. The SGR was determined using the formula SGR = 100 × ln [(W_t_ - W_0_) / t], where W_t_ represents the final shrimp weight, W_0_ is the initial weight, and t denotes the rearing period.

### Shrimp immune response

The analysis of shrimp immune response parameters involved observations of total hemocyte count (THC), phagocytosis activity (PA), phenoloxidase activity (PO), and respiratory burst activity (RB). THC observations were carried out according to Hamsah et al. [27] by taking body fluids from shrimp with a ratio of shrimp weight to anticoagulant (3.8 % sodium citrate, 30 mM of trisodium citrate, 338 mM of sodium chloride, 115 mM of glucose, 10 mM of EDTA, pH 7.0) of 1:3, which were then homogenized and observed using a hemacytometer under a microscope with a 100 × magnification. PA observations were carried out following Anderson and Siwicki [28] by incubating 100 µL of hemocytes mixed with 25 µL of *Staphylococcus aureus* suspension (10^7^ CFU/mL) for 20 min, which was then fixed with absolute methanol for five minutes, air-dried, and stained with Giemsa for 20 min. The prepared PA samples were observed using a microscope with a 400 × magnification. Meanwhile, PO observations were carried out following Liu and Chen [29], who observed dopachrome formation using an l-DOPA reagent and a spectrophotometer with a wavelength of 490 nm. Finally, RB observations referred to the method of Cheng et al. [30], who observed the reduction of nitroblue tetrazolium (NBT) per 10 µL of hemolymph using a spectrophotometer with a wavelength of 630 nm. The immune responses were observed on the 1st, 11th, and 21st days after the challenge test.

### Statistical analyses

Data analysis was conducted using analysis of variance (ANOVA) in SPSS version 25 and Tukey’s test with a 95 % confidence interval to see if significant differences were detected. Each treatment was conducted with three replicates to ensure statistical reliability. The Kolmogorov–Smirnov and Levene tests were used to evaluate the homogeneity and normality of the data, respectively.

## Results

### Water quality

Based on observations, the water quality, DO, pH, and salinity were in the optimal ranges for shrimp culture: DO 4.7–6.2 mg/L, pH 6.9–7.9, salinity 31–34 g/L. The total ammoniacal nitrogen (TAN), nitrite, and nitrate values for the biofloc treatments were lower than those for the control treatments (Table 2). The floc volumes were maintained according to the floc volume treatments.

**Table 2:** Water quality in the white shrimp rearing media with different biofloc volumes and challenge from *V. parahaemolyticus.*

Treatments	TAN (mg/L)	Nitrite (mg/L)	Nitrate (mg/L)	Biofloc Volume (mL/L)	TSS (mg/L)
Negative Control	0.02–0.04	0.29–0.38	0.76–0.86	0	0.0–42.3
Positive Control	0.02–0.03	0.25–0.30	0.68–0.73	0	0.0–52.2
Biofloc 5 mL/L	0.02–0.03	0.18–0.19	0.48–0.65	4.9–5.5	111.0–121.5
Biofloc 10 mL/L	0.01–0.02	0.21–0.26	0.73–0.86	9.5–10.8	141.3–151.8
Biofloc 15 mL/L	0.01–0.02	0.19–0.24	0.57–0.70	14.5–15.7	192.2–212.3

Notes: TAN: total ammoniacal nitrogen, TSS: total suspended solid.

### The effect of different biofloc densities on *vibrio* density and biofilm formation

Table 3 demonstrates that biofloc suspension was able to reduce the density of *V. parahaemolyticus* by up to 46.36 % in a biofloc suspension volume of 15 mL/L, which was significantly higher than in the 5 and 10 mL/L biofloc suspension volumes (*p* < 0.05). Furthermore, biofloc filtrate was also able to reduce the density of *V. parahaemolyticus* by 10.22–15.21 % and did not show any significant differences between biofloc treatments.

**Table 3:** *V. parahaemolyticus* density with the addition of biofloc suspension and biofloc filtrate.

Treatments	*V. parahaemolyticus* Density (CFU/mL)	Bacterial Inhibition ( %)
Control	9.396 ± 0.019^d^	–
Biofloc suspension 5 mL/L	5.992 ± 0.059^b^	36.23 ± 0.99^b^
Biofloc suspension 10 mL/L	5.935 ± 0.118^ab^	36.85 ± 2.25^b^
Biofloc suspension 15 mL/L	5.040 ± 0.020^a^	46.36 ± 0.20^c^
Biofloc filtrate 5 mL/L	8.437 ± 0.146^c^	10.22 ± 2.74^a^
Biofloc filtrate 10 mL/L	8.391 ± 0.070^c^	10.70 ± 1.13^a^
Biofloc filtrate 15 mL/L	7.967 ± 0.032^c^	15.21 ± 0.33^a^

Different superscript letters following the mean (± standard error) in the same column indicate significant differences (*p* < 0.05). Each treatment has three replications.

Biofloc suspension and biofloc filtrate significantly inhibited *V. parahaemolyticus* biofilm activity compared to the controls (*p* < 0.05). The highest inhibition activity (45.16 %) was observed in the 15 mL/L treatment, which was significantly higher than the inhibition activity observed in the 5 mL/L treatment (*p* < 0.05) (Table 4).

**Table 4:** *V. parahaemolyticus* biofilm inhibition with the addition of biofloc suspension and biofloc filtrate.

Treatments	Biofilm Activity (O.D 650 nm)	Biofilm Inhibition ( %)
Control	0.0810 ± 0.0008^c^	–
Biofloc suspension 5 mL/L	0.0530 ± 0.006^a^	38.61 ± 3.42^ab^
Biofloc suspension 10 mL/L	0.0483 ± 0.0009^a^	44.07 ± 2.71^b^
Biofloc suspension 15 mL/L	0.0473 ± 0.007^a^	45.16 ± 3.19^b^
Biofloc filtrate 5 mL/L	0.0663 ± 0.0012^b^	23.20 ± 4.09^a^
Biofloc filtrate 10 mL/L	0.0587 ± 0.0003^ab^	31.98 ± 4.21^ab^
Biofloc filtrate 15 mL/L	0.0527 ± 0.0007^a^	39.01 ± 3.25^ab^

Different superscript letters following the mean (± standard error) in the same column indicate significant differences (*p* < 0.05). Each treatment has three replications.

### Shrimp growth performance

The evaluation of shrimp growth performance, as indicated by the Wt and SGR parameters, revealed a statistically significant improvement in the biofloc system treatment compared to the control (*P* < 0.05). However, no significant differences were observed among the various biofloc density treatments (Table 5).

**Table 5:** Growth performance of shrimp reared in media with different biofloc densities and challenged with *V. parahaemolyticus.*

Treatments	Parameters			
	W_0_ (g)	W_t_ (g)	ΔW (g)	SGR ( %)
Negative Control	0.523 ± 0.147^a^	1.005 ± 0.005^ab^	0.482 ± 0.014^a^	3.58 ± 0.47^b^
Positive Control	0.550 ± 0.157^a^	0.993 ± 0.006^a^	0.463 ± 0.040^a^	2.36 ± 0.24^a^
Biofloc 5 mL/L	0.527 ± 0.170^a^	1.017 ± 0.006^bc^	0.049 ± 0.035^a^	4.71 ± 0.19^c^
Biofloc 10 mL/L	0.527 ± 0.153^a^	1.020 ± 0.005^c^	0.493 ± 0.023^a^	5.04 ± 0.57^c^
Biofloc 15 mL/L	0.513 ± 0.163^a^	1.024 ± 0.002^c^	0.510 ± 0.045^a^	5.53 ± 0.23^c^

Different superscript in the same parameter indicate significant differences (*p* < 0.05). Each treatment has three replications.

### Shrimp immune response and post-challenge survival

**Fig. 1:**
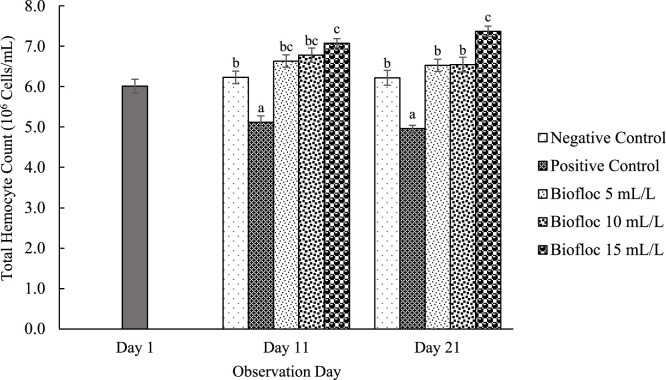
Total hemocyte count of white shrimp reared in media with different biofloc densities and challenged with *V. parahaemolyticus.* Different letters above the bars on the same observation day indicate significant differences (*p* < 0.05). Each treatment has three replications.

**Fig. 2:**
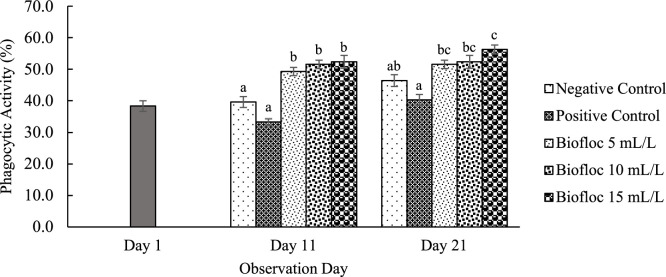
Phagocytic activity of white shrimp reared in media with different biofloc densities and challenged with *V. parahaemolyticus.* Different letters above the bars on the same observation day indicate significant differences (*p* < 0.05). Each treatment has three replications.

**Fig. 3:**
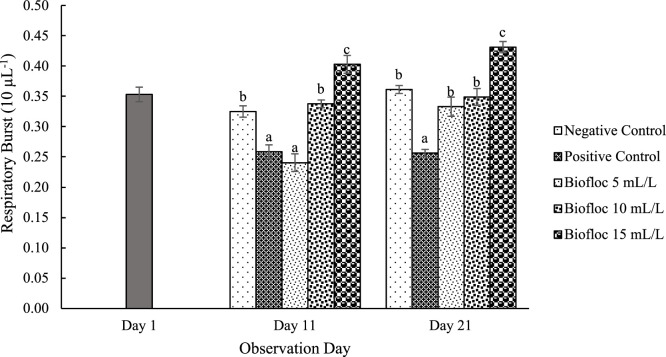
Respiratory burst of white shrimp reared in media with different biofloc densities and challenged with *V. parahaemolyticus.* Different letters above the bars on the same observation day indicate significant differences (*p* < 0.05). Each treatment has three replications.

**Fig. 4:**
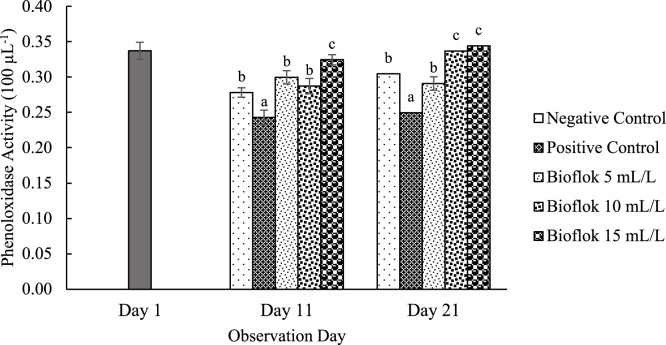
Phenoloxidase activity of white shrimp reared in media with different biofloc densities and challenged with *V. parahaemolyticus.* Different letters above the bars on the same observation day indicate significant differences (*p* < 0.05). Each treatment has three replications.

All floc volume treatments increased THC on the 11th and 21st days of observation compared to the controls. On day 21, the THC values for the 15 mL/L floc volume treatment were significantly higher than those for the other floc volume treatments (Fig. 1). Furthermore, the phagocytic activity in all different volume biofloc treatments was higher than that in the controls on the 11th and 21st days of observation, but the difference was not significant (Fig. 2). The 15 mL/L floc volume treatment showed the highest respiratory burst activity, which was significantly different (*p* < 0.05) from the values in the other biofloc treatments and controls on both the 11th and 21st days. On the 11th day of treatment, the 5 mL/L biofloc volume treatment did not show any significant difference from the controls (Fig. 3). The application of biofloc in different volumes could increase the phenoloxidase activity. The biofloc volume of 15 mL/L showed the highest phenoloxidase activity, which was significantly different from the values for the control and 5 mL/L biofloc volume treatments (*p* < 0.05; Fig. 4). Furthermore, the *V. parahaemolyticus* density in the bodies of the shrimp reared with the 15 mL/L biofloc volume treatment was significantly lower than the value for the positive control (Table 6).

**Table 6:** *V. parahaemolyticus* density in the rearing media and the bodies of the shrimp reared in the biofloc system with different volumes and challenge from *V. parahaemolyticus.*

Treatments	*V. parahaemolyticus* density	
	Water (Log CFU/mL)	Body (Log CFU/g)
Negative Control	–	–
Positive Control	4.83 ± 0.17^a^	4.87 ± 0.14^b^
Biofloc 5 mL/L	4.54 ± 0.28^a^	4.31 ± 0.02^ab^
Biofloc 10 mL/L	4.36 ± 0.13^a^	4.49 ± 0.06^ab^
Biofloc 15 mL/L	4.33 ± 0.02^a^	4.05 ± 0.09^a^

Different superscript letters following the mean (± standard error) in the same column indicate significant differences (*p* < 0.05). Each treatment has three replications.

**Fig. 5:**
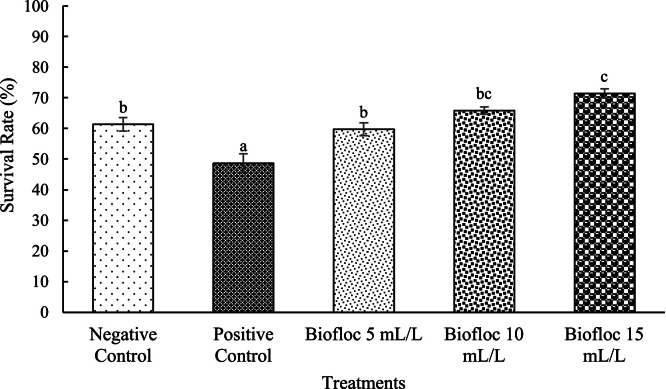
The survival rates of white shrimp reared in media with different biofloc densities and challenged with *V. parahaemolyticus.* Different letters above the bars on the same observation day indicate significant differences (*p* < 0.05). Each treatment has three replications.

The survival rates observed for all biofloc volume treatments were significantly higher than the rate for the positive control (*p* < 0.05). To be precise, the 15 mL/L biofloc volume treatment showed the highest survival rate, which was significantly different from the survival rate for the 5 mL/L biofloc volume treatment (*p* < 0.05) (Fig. 5). Additionally, regression analysis was conducted to determine the optimal biofloc concentration, yielding the equation: *y* = −0.0005x² + 0.0229x + 0.4897, with an R² value of 0.8897. Based on this regression model, the best biofloc concentration is predicted to be at a biofloc volume of 23 mL/L, with the best survival rate estimated at approximately 75 % under the study's rearing conditions

## Discussion

Biofloc technology improves water quality in aquaculture systems by reducing harmful ammonia and nitrite levels, both of which are detrimental to shrimp health. This is achieved through the conversion of nitrogenous waste into microbial biomass, which also serves as a biocontrol agent against pathogenic bacteria [31,32]. The current study demonstrated that biofloc effectively reduced *V. parahaemolyticus* density and inhibited biofilm formation *in vitro*, while also decreasing *Vibrio* loads in shrimp, enhancing the immune response, and improving resistance against *V. parahaemolyticus* infection. Additionally, biofloc contributed to improved water quality by lowering nitrogen concentrations in the system.

The antimicrobial effects of biofloc may be attributed to its production of extracellular compounds that interfere with bacterial communication (quorum sensing), thereby reducing the expression of bacterial virulence factors. Kumar et al. [33] demonstrated that biofloc can alter the phenotypic characteristics of *V. parahaemolyticus*, reducing its virulence. Similarly, the current study confirmed that biofloc could decrease both the density and biofilm formation of *V. parahaemolyticus* [16].

In this study, both biofloc suspension and its extracellular products (filtrate) inhibited the growth and biofilm formation of *V. parahaemolyticus*. While biofloc suspension was more effective due to competition for nutrients and space, the filtrate still reduced *V. parahaemolyticus* density by approximately 15 % compared to the controls, indicating the presence of antimicrobial properties in biofloc’s extracellular products. Ju et al. [34] identified several bioactive compounds produced by biofloc, such as bromophenol, carotenoids, chlorophyll, and amino acids, which have been shown to suppress the virulence functions in *V. parahaemolyticus*. Bromophenol, in particular, has demonstrated antibacterial activity against a range of pathogens, including *V. parahaemolyticus* [35]. In addition, among the vast diversity of bacterial species, the genus *Bacillus* is widely recognized for its remarkable ability to produce a broad range of enzymes at high levels. This group of bacteria stands out due to its efficiency and versatility in synthesizing various enzymes, which are essential for numerous industrial and biotechnological applications. The capability of *Bacillus* species to thrive in diverse environments and their proficiency in secreting extracellular enzymes contribute significantly to their reputation as the leading producers of enzymes among bacteria [36].

The reduction in biofilm formation observed in this study is likely mediated by quorum sensing disruption, either through direct interference by microbial aggregates within the biofloc or through the extracellular products produced by biofloc. Quorum sensing is a key mechanism involved in biofilm formation, virulence, and resistance to antimicrobials [[37], [38], [39], [40]].

The improved growth performance observed in shrimp reared under the biofloc system aligns with previous reports highlighting the advantages of biofloc technology in aquaculture. Biofloc systems enhance nitrogen utilization by converting waste feed and metabolic byproducts into microbial biomass, which serves as a readily available natural feed source, thereby supporting better nutrient cycling and growth [[41], [42]]. In the present study, shrimp maintained in biofloc conditions exhibited significantly higher specific growth rates (SGR) and final weights (Wt) compared to those in the control treatment, regardless of biofloc density variations. Notably, this superior growth performance persisted even after challenge exposure to *V. parahaemolyticus*, indicating the robustness of the biofloc system in maintaining shrimp growth under pathogenic stress.

Biofloc also stimulates immune responses in shrimp, with its microbial aggregates recognized by the pathogen-associated molecular pattern system, leading to increased hemocyte counts and enhanced cellular and humoral immune responses [43]. These immune responses include phagocytic activity, encapsulation, nodule formation, and activation of the prophenoloxidase (proPO) system, all of which play crucial roles in the defense against *V. parahaemolyticus* infection [44]. In this study, the shrimp reared in the biofloc system exhibited significantly higher hemocyte counts and phagocytic activity compared to the controls, with the highest immune stimulation observed in the treatment with the biofloc volume of 15 mL/L. This is consistent with the findings by Ferreira et al. [45], which demonstrated the immune-boosting effects of biofloc.

Additionally, the humoral immune response, mediated by phenoloxidase (PO) activity, plays a critical role in pathogen inactivation and the prevention of pathogen spread by promoting melanin formation, which helps neutralize invading microbes [46]. The production of reactive oxygen intermediates (ROIs), including hydrogen peroxide (H₂O₂), singlet oxygen (¹O₂), and hydroxyl radicals (OH), further contributes to pathogen elimination [47,48]. The significantly higher PO and respiratory burst (RB) activity observed in the 15 mL/L biofloc volume treatment suggests an enhanced protective effect against *V. parahaemolyticus* infection compared to lower biofloc volumes (5 and 10 mL/L).

Shrimp survival rates post-challenge were significantly higher in the biofloc-treated groups compared to the controls, likely due to reduced virulence factor expression in *V. parahaemolyticus* and improved shrimp immune responses. The highest survival rate, observed in the 15 mL/L floc volume treatment (71.4 ± 1.5 %), was significantly higher than that of the 5 mL/L floc volume group (59.7 ± 2.1 %). These findings are consistent with previous studies that reported improved shrimp survival in biofloc systems challenged with *V. parahaemolyticus* [19,49]. The reduced bacterial load in shrimp bodies further supports the protective effects of biofloc at this optimal density.

## Conclusion

In conclusion, biofloc suspension and its extracellular products, with *Bacillus* sp. NP5 supplementation, are effective in reducing *V. parahaemolyticus* density and biofilm formation. Biofloc at a density of 15 mL/L provides the highest protection, enhancing shrimp immune responses (hemocyte count, phagocytic activity, respiratory burst, and phenoloxidase activity) and resistance to *V. parahaemolyticus* infection. While no significant difference in growth performance was observed among the biofloc density treatments, the biofloc system overall showed better growth performance compared to the control groups. Additionally, biofloc improves water quality and reduces nitrogenous waste in the culture environment. Therefore, maintaining biofloc density at 15 mL/L is recommended to optimize the benefits of biofloc in shrimp aquaculture systems. Future studies should evaluate biofloc densities higher than 15 mL/L, especially considering the regression analysis in this study, which predicts the optimal biofloc density to be around 23 mL/L. Further research is necessary to confirm this prediction and explore the potential advantages of higher biofloc concentrations for enhancing shrimp health, immunity, and overall aquaculture performance.

## Animal research

This research involved animals and was approved by the Animal Care and Use Committee (ACUC) (Registration Number: 194–2021 IPB).

## CRediT authorship contribution statement

**Muhamad Gustilatov:** Writing – original draft, Visualization, Software, Resources, Project administration, Methodology, Funding acquisition, Formal analysis, Data curation, Conceptualization. **Widanarni:** Writing – review & editing, Validation, Supervision, Methodology, Investigation, Funding acquisition, Formal analysis, Conceptualization. **Julie Ekasari:** Writing – review & editing, Validation, Supervision, Investigation. **Pande Gde Sasmita Julyantoro:** Writing – review & editing, Validation, Supervision, Investigation, Formal analysis, Conceptualization. **Diana Elizabeth Waturangi:** Writing – review & editing, Validation, Supervision, Formal analysis, Conceptualization. **Sukenda:** Writing – review & editing, Validation, Supervision, Formal analysis. **Febrina Losiana Silalahi:** Writing – original draft, Software, Methodology, Formal analysis, Data curation.

## Declaration of competing interest

The authors declare that they have no known competing financial interests or personal relationships that could have appeared to influence the work reported in this paper.

## Data Availability

The data underlying this article will be shared on reasonable request to the corresponding author.

## References

[1] FAO [Food and Agricultural Organization of the United Nations] (2024).

[2] Tran L., Nunan L., Redman R.M., Mohney L.L., Pantoja C.R., Fitzsimmons K., Lightner D.V. (2013). Determination of the infectious nature of the agent of acute hepatopancreatic necrosis syndrome affecting penaeid shrimp. Dis. Aquat. Org..

[3] Valente C.D.S., Wan A.H. (2020). *Vibrio* and major commercially important vibriosis diseases in decapod crustaceans. J Invertbr Pathol.

[4] Tang K.F.J., Bondad-Reantaso M.G. (2019). Impacts of acute hepatopancreatic necrosis disease on commercial shrimp aquaculture. Rev. Sci. Tech..

[5] Chan M.W.H., Ali A., Ullah A., Mirani Z.A., Balthazar-Silva D. (2021). A size-dependent bioaccumulation of metal pollutants, antibacterial and antifungal activities of Telescopium telescopium, Nerita albicilla and Lunella coronate. Environ. Toxicol. Pharmacol..

[6] Ben-Yaakov R., Salomon D. (2019). The regulatory network of *Vibrio parahaemolyticus* type VI secretion system 1. Environ. Microb..

[7] Li L., Meng H., Dan G., Yang L., Mengdie J. (2019). Molecular mechanisms of *vibrio parahaemolyticus* pathogenesis. Microbiol. Res..

[8] Qiu Y., Hu L., Yang W., Yin Z., Zhou D., Yang H., Zhang Y. (2020). The type VI secretion system 2 of *Vibrio parahaemolyticus* is regulated by QsvR. Microb. Pathog..

[9] Lin S.J., Huang J.Y., Le P.T., Lee C.T., Chang C.C., Yang Y.Y., Wang H.C. (2022). Expression of the AHPND toxins PirAvp and PirBvp is regulated by components of the *Vibrio parahaemolyticus* quorum sensing (QS) system. Int. J. Mol. Sci..

[10] Zhang Y., Hu L., Qiu Y., Osei-Adjei G., Tang H., Zhang Y., Zhang R., Sheng X., Xu S., Yang W. (2019). QsvR integrates into quorum sensing circuit to control *Vibrio parahaemolyticus* virulence. Environ. Microbiol..

[11] Zhao J., Chen M., Quan C.S., Fan S.D. (2015). Mechanisms of quorum sensing and strategies for quorum sensing disruption in aquaculture pathogens. J. Fish Dis..

[12] Kumar V., Roy S., Behera B.K., Bossier P., Das B.K. (2021). Acute hepatopancreatic necrosis disease (AHPND): virulence, pathogenesis, and mitigation strategies in shrimp aquaculture. Toxins (Basel).

[13] Avnimelech Y. (2009).

[14] Huang H.H., Liao H.M., Lei Y.J., Yang P.H. (2022). Effects of different carbon sources on growth performance of *Litopenaeus vannamei* and water quality in the biofloc system in low salinity. Aquaculture.

[15] Crab R., Lambert A., Defoirdt T., Bossier P., Verstraete W. (2010). The application of bioflocs technology to protect brine shrimp (*Artemia franciscana*) from pathogenic *Vibrio harveyi*. J. Appl. Microbiol..

[16] Gustilatov M., Widanarni, Ekasari J., Pande G.S.J. (2022). Protective effects of the biofloc system in Pacific white shrimp (Penaeus vannamei) culture against pathogenic Vibrio parahaemolyticus infection. Fish. Shellfish. Immunol..

[17] Widanarni W., Gustilatov M., Ekasari J., Julyantoro P.G.S., Waturangi D.E., Sukenda S. (2024). Unveiling the positive impact of biofloc culture on Vibrio parahaemolyticus infection of Pacific white shrimp by reducing quorum sensing and virulence gene expression and enhancing immunity. J. Fish Dis..

[18] Aguilera-Rivera D., Prieto-Davó A., Escalante K., Chávez C., Cuzon C., Gaxiola G. (2014). Probiotic effect of FLOC on Vibrios in the Pacific white shrimp *Litopenaeus vannamei*. Aquaculture.

[19] Hostins B., Wasielesky W., Decamp O.C., Bossier P., De Schryver P. (2019). Managing input C/N ratio to reduce the risk of Acute Hepatopancreatic Necrosis Disease (AHPND) outbreaks in biofloc systems – A laboratory study. Aquaculture.

[20] De Schryver P., Crab R., Defoirdt T., Boon N., Verstraete W. (2008). The basics of bio-flocs technology: the added value for aquaculture. Aquaculture.

[21] Li X., Li S., Yu Y., Zhang X., Xiang J., Li F. (2022). The immune function of a NLR-like gene, LvNLRPL1, in the Pacific whiteleg shrimp *Litopenaeus vannamei*. Dev. Comp. Immunol..

[22] Gustilatov M., Widanarni W., Ekasari J., Julyantoro P.G.S., Waturangi D.E. (2023). Biofloc system supplemented by Pseudoalteromonas piscicida 1Ub protects the Pacific white shrimp Penaeus vannamei from Vibrio parahaemolyticusinfection. Aquac. Fisheries.

[23] APHA (2005).

[24] Raissa G., Waturangi D.E., Wahjuningrum D. (2020). Screening of antibiofilm and anti-quorum sensing activity of Actinomycetes isolates extracts against aquaculture pathogenic bacteria. BMC Microbiol..

[25] Madigan M.T., Martinko J.M., Parker J. (2006).

[26] Liu Y., Xing R., Liu S., Qin Y., Li K., Yu H., Li P. (2019). Effects of chitooligosaccharides supplementation with different dosages, molecular weights and degrees of deacetylation on growth performance, innate immunity and hepatopancreas morphology in Pacific white shrimp (*Litopenaeus vannamei*). Carbohydr. Polym..

[27] Hamsah H., Widanarni W., Alimuddin A., Yuhana M., Junior M.Z., Hidayatullah D. (2019). Immune response and resistance of Pacific white shrimp larvae administered probiotic, prebiotic, and synbiotic through the bio-encapsulation of *Artemia* sp. Aquac Int.

[28] Anderson P., Siwicki A.K. (1993). Second Symposium on Diseases in Asia Aquaculture “Aquatic Animal Health and The Environmental.

[29] Liu C.H., Chen J.C. (2004). Effect of ammonia on the immune response of white shrimp *Litopenaeus vannamei* and its susceptibility to *Vibrio alginolyticus*. Fish Shellfish Immunol..

[30] Cheng W., Hsiao I., Hsu C., Chen J. (2004). Change in water temperature on the immune response of Taiwan abalone *Haliotis diversicolor supertexta* and its susceptibility to *Vibrio parahaemolyticus*. Fish Shellfish Immunol..

[31] Ekasari J., Azhar M.H., Surawidjaja E.H., Nuryati S., De Schryver P., Bossier P. (2014). Immune response and disease resistance of shrimp fed biofloc grown on different carbon source. Fish Shellfish. Immunol..

[32] Panigrahi A., Saranya C., Sundaram M., Kannan S.R.V., Das R.R., Kumar R.S., Rajesh P., Otta S.K. (2018). Carbon: nitrogen (C) ratio level variation influences microbial community of the system and growth as well as immunity of shrimp (*Litopenaeus vannamei*) in biofloc-based culture system. Fish Shellfish Immunol..

[33] Kumar V., Wille M., Lourenço T.M., Bossier P. (2020). Biofloc-based enhanced survival of *Litopenaeus vannamei*upon AHPND-causing *vibrio parahaemolyticus* challenge is partially mediated by reduced expression of its virulence genes. Front. Microbiol..

[34] Ju Z.Y., Forster I., Conquest L., Dominy W., Kuo W.C., Horgen F.D. (2008). Determination of microbial community structures of shrimp floc cultures by biomarkers and analysis of floc amino acid profiles. Aquac Res.

[35] Kurhekar J.V., Gupta V.K. (2020). Phytochemicals As Lead Compounds for New Drug Discovery.

[36] Naveed M., Wen S., Chan M.W.H., Wang F., Yin X., Xu B., Ullah A. (2024). Expression of BSN314 lysozyme genes in *Escherichia coli* BL21: a study to demonstrate microbicidal and disintegarting potential of the cloned lysozyme. Braz. J. Microbiol..

[37] Liu J., Fu K., Wu C., Qin K., Li F., Zhou L. (2018). In-group” communication in marine *Vibrio*: a review of n-acyl homoserine lactones-driven quorum sensing. Front. Cell. Infect. Microbiol..

[38] Liu H., Li H., Wei H., Zhu X., Han D., Jin J., Yang Y., Xie S. (2019). Biofloc formation improves water quality and fish yield in a freshwater pond aquaculture system. Aquaculture.

[39] Milton D.L. (2006). Quorum sensing in vibrios: complexity for diversification. Int. J. Med. Microbiol..

[40] Packiavathy I.A.S.V., Sasikumar P., Pandian S.K., Veera Ravi A. (2013). Prevention of quorum-sensing-mediated biofilm development and virulence factors production in *Vibrio* spp. By curcumin. Appl. Microbiol. Biotechnol..

[41] Kumar V.S., Pandey P.K., Anand T., Bhuvaneswari G.R., Dhinakaran A., Kumar S. (2018). Biofloc improves water, effluent quality and growth parameters of *Penaeus vannamei* in an intensive culture system. J. Environ. Manag..

[42] Rajkumar M., Pandey P.K., Aravind R., Vennila A., Bharti V., Purushothaman C.S. (2016). Effect of different biofloc system on water quality, biofloc composition and growth performance in *Litopenaeus vannamei* (Boone, 1931). Aquac Res.

[43] Kim S.K., Pang Z., Seo H.C., Cho Y.R., Samocha T., Jang I.K. (2014). Effect of bioflocs on growth and immune activity of Pacific white shrimp, *Litopenaeus vannamei* postlarvae. Aquac Res.

[44] Sahoo P.K., Das A., Mohanty B.K., Pilai B., Mohanty J. (2008). Dietary β-1,3 glucan improve the immunity and disease resistance of freshwater prawn *macrobrachium rosenbergii* challenged with *Aeromonas hydrophyla*. Aquac. Res..

[45] Ferreira G.S., Bolivar N.C., Pereira S.A., Guertler C., do Nascimento Vieira F., Mouriño J.L.P., Seiffert W.Q. (2015). Microbial biofloc as a source of probiotic bacteria for the culture of *Litopenaeus vannamei*. Aquaculture.

[46] Amparyup P., Charoensapsri W., Tassanakajon A. (2013). Prophenoloxidase system and its role in shrimp immune responses against major pathogens. Fish Shellfish Immunol..

[47] Munoz M., Cedeno R., Rodríguez J., van der Knaap W.P., Mialhe E., Bachere E. (2000). Measurement of reactive oxygen intermediate production in haemocytes of the penaeid shrimp, *Penaeus vannamei*. Aquaculture.

[48] Rodrıguez J., Moullac G.L. (2000). State of the art of immunological tools and health control of penaeid shrimp. Aquaculture.

[49] Sajali U.S.B.A., Atkinson N.L., Desbois A.P., Little D.C., Murray F.J., Shinn A.P. (2019). Prophylactic properties of biofloc-or Nile tilapia-conditioned water against *Vibrio parahaemolyticus* infection of whiteleg shrimp (*Penaeus vannamei*). Aquaculture.

